# Trends in obesity and diabetes across Africa from 1980 to 2014: an analysis of pooled population-based studies

**DOI:** 10.1093/ije/dyx078

**Published:** 2017-06-04

**Authors:** NCD Risk Factor Collaboration (NCD-RisC) – Africa Working Group, Andre Pascal Kengne, James Bentham, Bin Zhou, Nasheeta Peer, Tandi E Matsha, Honor Bixby, Mariachiara Di Cesare, Kaveh Hajifathalian, Yuan Lu, Cristina Taddei, Pascal Bovet, Catherine Kyobutungi, Charles Agyemang, Hajer Aounallah-Skhiri, Felix K Assah, Amina Barkat, Habiba Ben Romdhane, Queenie Chan, Nishi Chaturvedi, Albertino Damasceno, Hélène Delisle, Francis Delpeuch, Kouamelan Doua, Eruke E Egbagbe, Jalila El Ati, Paul Elliott, Reina Engle-Stone, Rajiv T Erasmus, Heba M Fouad, Dickman Gareta, Oye Gureje, Marleen Elisabeth Hendriks, Leila Houti, Mohsen M Ibrahim, Han C G Kemper, Japhet Killewo, Sudhir Kowlessur, Herculina S Kruger, Fatima Zahra Laamiri, Youcef Laid, Naomi S Levitt, Nuno Lunet, Dianna J Magliano, Bernard Maire, Yves Martin-Prevel, Sounnia Mediene-Benchekor, Mostafa K Mohamed, Charles K Mondo, Kotsedi Daniel Monyeki, Aya Mostafa, Martin Nankap, Ellis Owusu-Dabo, Tobias F Rinke de Wit, Olfa Saidi, Constance Schultsz, Aletta E Schutte, Idowu O Senbanjo, Jonathan E Shaw, Liam Smeeth, Eugène Sobngwi, Charles Sossa Jérome, Karien Stronks, Frank Tanser, Félicité Tchibindat, Pierre Traissac, Lechaba Tshepo, Fikru Tullu, Flora A M Ukoli, Bharathi Viswanathan, Alisha N Wade, Goodarz Danaei, Gretchen A Stevens, Leanne M Riley, Majid Ezzati, Jean Claude N Mbanya

**Keywords:** Diabetes, adiposity, body mass index, Africa, prevalence, trends

## Abstract

**Background:**

The 2016 *Dar Es Salaam Call to Action on Diabetes and Other non-communicable diseases (NCDs)* advocates national multi-sectoral NCD strategies and action plans based on available data and information from countries of sub-Saharan Africa and beyond. We estimated trends from 1980 to 2014 in age-standardized mean body mass index (BMI) and diabetes prevalence in these countries, in order to assess the co-progression and assist policy formulation.

**Methods:**

We pooled data from African and worldwide population-based studies which measured height, weight and biomarkers to assess diabetes status in adults aged ≥ 18 years. A Bayesian hierarchical model was used to estimate trends by sex for 200 countries and territories including 53 countries across five African regions (central, eastern, northern, southern and western), in mean BMI and diabetes prevalence (defined as either fasting plasma glucose of ≥ 7.0 mmol/l, history of diabetes diagnosis, or use of insulin or oral glucose control agents).

**Results:**

African data came from 245 population-based surveys (1.2 million participants) for BMI and 76 surveys (182 000 participants) for diabetes prevalence estimates. Countries with the highest number of data sources for BMI were South Africa (*n* = 17), Nigeria (*n* = 15) and Egypt (*n* = 13); and for diabetes estimates, Tanzania (*n* = 8), Tunisia (*n* = 7), and Cameroon, Egypt and South Africa (all *n* = 6). The age-standardized mean BMI increased from 21.0 kg/m^2^ (95% credible interval: 20.3–21.7) to 23.0 kg/m^2^ (22.7–23.3) in men, and from 21.9 kg/m^2^ (21.3–22.5) to 24.9 kg/m^2^ (24.6–25.1) in women. The age-standardized prevalence of diabetes increased from 3.4% (1.5–6.3) to 8.5% (6.5–10.8) in men, and from 4.1% (2.0–7.5) to 8.9% (6.9–11.2) in women. Estimates in northern and southern regions were mostly higher than the global average; those in central, eastern and western regions were lower than global averages. A positive association (correlation coefficient ≃ 0.9) was observed between mean BMI and diabetes prevalence in both sexes in 1980 and 2014.

**Conclusions:**

These estimates, based on limited data sources, confirm the rapidly increasing burden of diabetes in Africa. This rise is being driven, at least in part, by increasing adiposity, with regional variations in observed trends. African countries’ efforts to prevent and control diabetes and obesity should integrate the setting up of reliable monitoring systems, consistent with the World Health Organization’s Global Monitoring System Framework.

**Key Messages**

- This is the first detailed analysis of trends in adiposity and diabetes prevalence in Africa, both overall and by major geographical regions.
- We used validated methods to re-analyse and pool population-based sources with measured data on at least one diabetes biomarker and/or body weight and height, including over 250 African sources.
- Age-standardized mean body mass index and diabetes prevalence have steadily increased across Africa since 1980, tracking in general with the global averages.
- At any given time, estimates in northern Africa (driven by Egypt) and in southern Africa (driven by South Africa) appeared higher than the global average, whereas estimates for other regions were mostly lower.
- There was a weak association between change in gross domestic product and change in adiposity and diabetes prevalence, highlighting the coping challenges facing African countries, and the need for innovative approaches to diabetes and obesity prevention and control.

## Introduction

Despite a high burden of infectious diseases in Africa, non-communicable diseases (NCDs), including type 2 diabetes mellitus (hereafter referred to as ‘diabetes’), are rising steadily, contributing substantially to morbidity and mortality. This is partly driven by a growing proportion of elderly people.^1^ Furthermore, economic growth and accompanying rapid urbanization have led to a burgeoning wealthier middle class, possibly with shifts in diet and physical activity contributing to the development of obesity. This, with a genetic predisposition and epigenetic changes,^2^^,^^3^ increases the risk of diabetes.^4^

Healthcare systems in Africa are now facing new disease burdens they are ill-equipped to manage. A number of NCD initiatives informed by global resolutions, including disease-specific initiatives such as for diabetes in Africa,^5–8^ have been developed.^4^^,^^9^ However, tangible responses from national and international stakeholders have been slow, and have largely overlooked adiposity and the unhealthy food environment. Driven by maternal, newborn and child health (MNCH), the traditional focus of investment in preventive health has been on undernutrition and food insecurity.

To highlight the increasing diabetes prevalence and the key contributory role played by obesity requires local data. Furthermore, despite the strong link between adiposity and diabetes, efforts to co-examine the burden of the two conditions have been limited. Here, we estimate trends in age-standardized mean body mass index (BMI) and diabetes prevalence across Africa from 1980 to 2014. We aimed to understand (i) the extent and quality of available data, (ii) differences in patterns by regions and sex and (iii) the association of adiposity with diabetes prevalence.

## Methods

The full methods of data collection, processing and analysis in the NCD Risk Factor Collaboration are available elsewhere^10^^,^^11^ and in the Supplementary Textbox 1 (available as Supplementary data at *IJE* online). We estimated trends for mean BMI and diabetes prevalence from 1980 to 2014, in ≥18-year-old adults across 53 countries organized into five regions (Supplementary Table 1, available as Supplementary data at *IJE* online).

We included data sources representative of national, sub-national or community populations, with measured weight and height or at least one of the following bio-markers: fasting plasma glucose (FPG), oral glucose tolerance test (OGTT) or glycosylated haemoglobin (HbA_1c_). We converted diabetes measured using different biomarkers and definitions to a common definition of FPG ≥ 7.0 mmol/l or a history of diabetes with ongoing treatment, as described previously.^11^ Analyses were done separately for men and women, using a statistical model described and validated previously.^10^^,^^11^ The model had a hierarchical structure in which estimates for each country and year were informed by its own data, data from other years in the same country and data in other countries in the same region. The model also accounted for non-linear time trends and age associations. The reported credible intervals (CrIs) represent the 2.5^th^-97.5^th^ percentiles of the posterior distributions. Estimates were standardized to the World Health Organization (WHO) standard population.

## Results

### Data available

**Figure 1:**
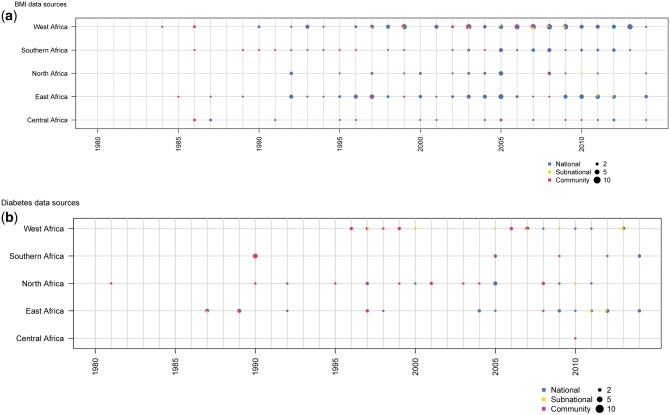
Number of data sources available for body mass index and diabetes by African region and year.

The analysis used data from 245 population-based surveys encompassing 1.2 million participants aged ≥ 18 years for BMI estimates, and 76 surveys (182 000 participants) for diabetes estimates.^10^^,^^11^ These data were collected between 1984 and 2014 in 50 countries (94% of African countries) for BMI, and between 1981 and 2014 in 32 countries (60% of African countries) for diabetes (Figure 1).^10^^,^^11^

**Figure 2:**
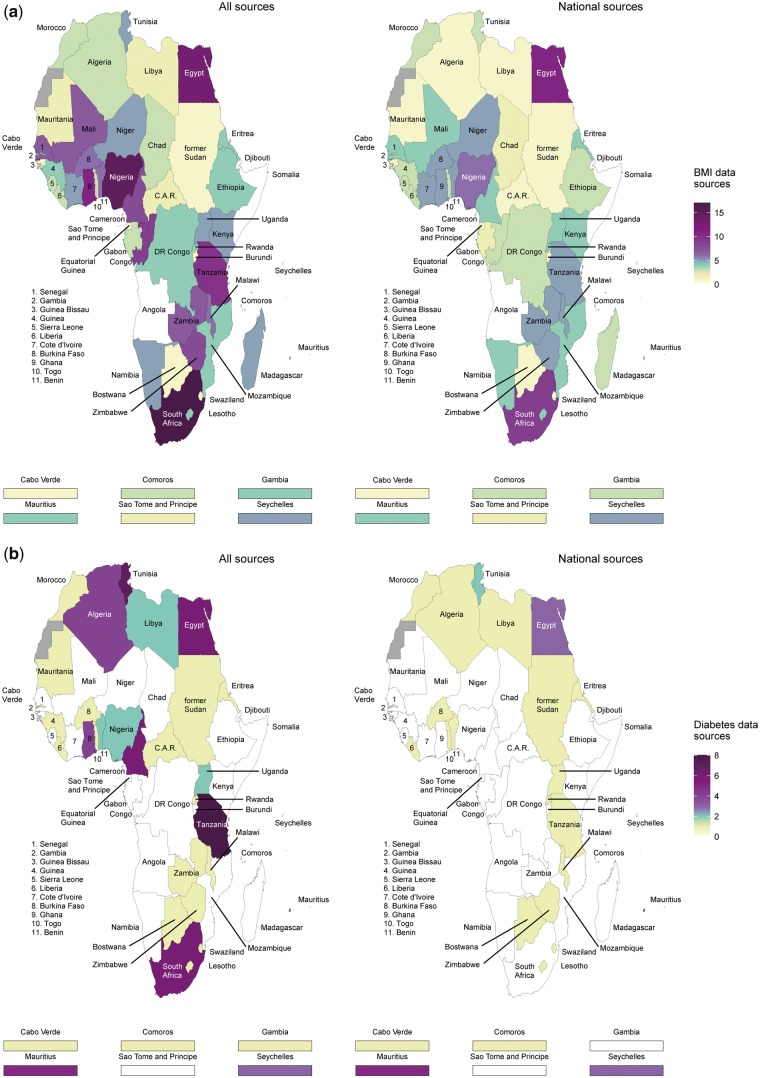
Number of data sources available for body mass index and diabetes by country in Africa.

The distribution of surveys per region for BMI estimates was as follows: 19 surveys (55 000 participants) for central Africa, 68 (325 000) for eastern, 26 (170 000) for northern, 36 (299 000) for southern and 96 (394 000) for western Africa (Figure 1a). South Africa (*n* = 17), Nigeria (*n* = 15) and Egypt (*n* = 13) had the highest number of data points (regardless of sources), whereas Angola, Djibouti and Somalia had none (Figure 2).

Equivalent figures for diabetes estimates were one survey (3600 participants) for central Africa, 24 (77 000) for eastern, 20 (43 000) for northern, 10 (14 000) for southern and 21 (45 000) for western Africa (Figure 1b). Tanzania (*n* = 8), Tunisia (*n* = 7), and Cameroon, Egypt and South Africa (all *n* = 6) had the highest number of data points. No data were available from 21 countries: Angola, Burundi, Chad, Congo, Cote d'Ivoire, Djibouti, DR Congo, Equatorial Guinea, Ethiopia, Gabon, Guinea Bissau, Kenya, Madagascar, Mali, Mozambique, Namibia, Niger, Sao Tome and Principe, Senegal, Sierra Leone, Somalia (Figure 2).

Regarding the representativeness of data sources, 173 surveys (1.1 million participants) for BMI and 32 surveys (104 000 participants) for diabetes prevalence estimates had national coverage, whereas the remainder were sub-national or community-based (Figure 1); 76 data sources (203 000 participants) for BMI and 28 data sources (60 000 participants) for diabetes (Figure 1) were collected prior to 2000. These data were augmented with 1453 sources for BMI and 675 for diabetes from other regions. This helped the fitting of features, such as age patterns and the effects of covariates through the hierarchical structure, which also influenced trends.

### Obesity trends

**Figure 3:**
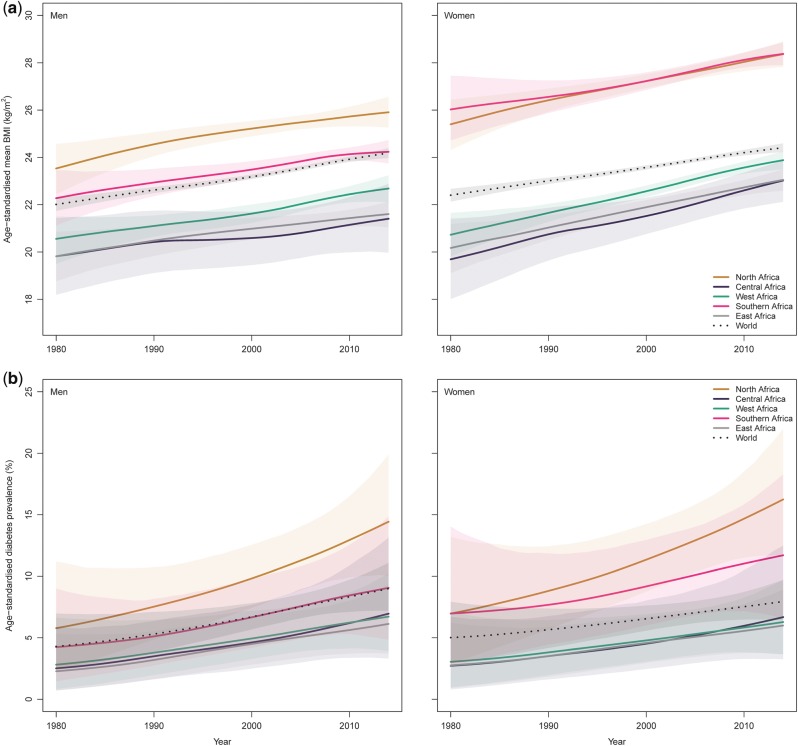
Trends in age-standardized mean body mass index and age-standardized diabetes prevalence by sex and region in Africa.

From 1980 to 2014, the age-standardized mean BMI increased from 21.0 kg/m^2^ (95% CrI 20.3–21.7) to 23.0 kg/m^2^ (22.7–23.3) in men, and from 21.9 kg/m^2^ (21.3–22.5) to 24.9 kg/m^2^ (24.6–25.1) in women. Mean BMI increased over time across all regions, paralleling the global average; however there were suggestions, over recent years, of steeper curves in women compared with worldwide trends (Figure 3a). In both men and women, the lowest mean BMI, over time, was usually recorded in central Africa; whereas the highest, for men, was recorded in northern Africa, and, for women, in northern and southern Africa.

**Figure 4:**
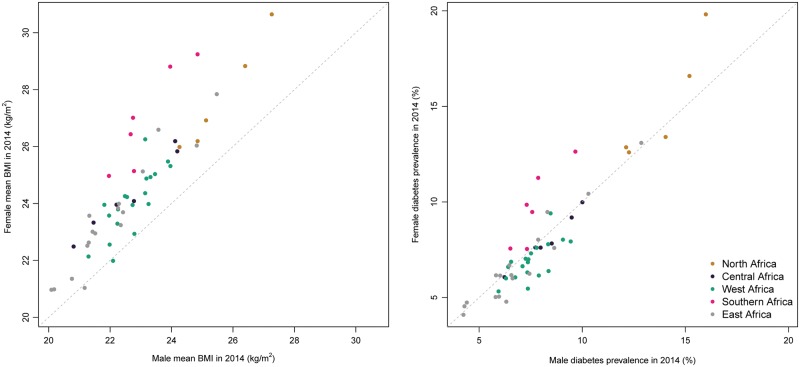
Scatterplot of male versus female age-standardized mean body mass index and age-standardized diabetes prevalence by African region in 2014.

**Figure 5:**
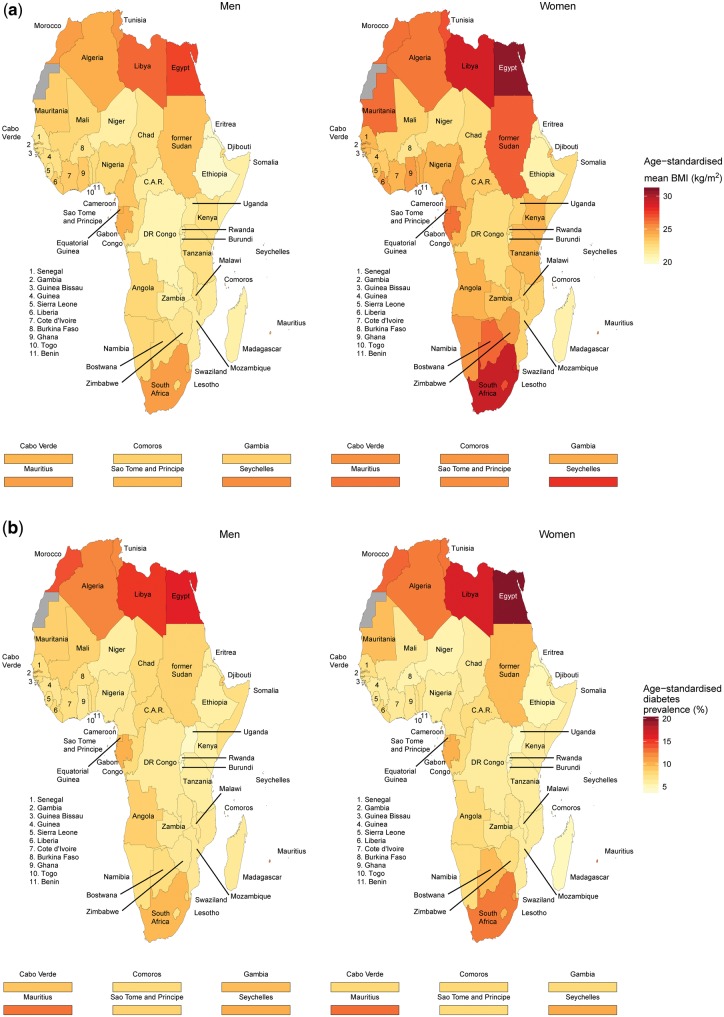
Age-standardized mean body mass index and age-standardized diabetes prevalence by sex and country in Africa in 2014.

Mean BMI was higher than the global average in northern and southern Africa, and lower in central, eastern and western Africa. In many cases, CrIs around mean estimates overlapped across regions and with that for the global mean, due to a combination of limited available data and the similarity of the estimates. A scatterplot of mean BMI in men versus women in 2014 is shown in Figure 4. Across the five regions, mean BMI appeared to be mostly higher in women than in men. Per country, mean BMI in 2014 ranged from 20.1 kg/m^2^ (19.2–21.0) in Ethiopia to 27.3 kg/m^2^ (26.5–28.1) in Egypt in men, and from 21.0 kg/m^2^ in Eritrea, Ethiopia and Madagascar to 30.6 kg/m^2^ (30.0–31.2) in Egypt in women (Figure 5a; and Supplementary Table 2, available as Supplementary data at *IJE* online).

### Diabetes prevalence trends

Age-standardized diabetes prevalence increased, from 1980 to 2014, from 3.4% (1.5–6.3) to 8.5% (6.5–10.8) in men, and from 4.1% (2.0–7.5) to 8.9% (6.9–11.2) in women. Across regions and in both sexes, age-standardized diabetes prevalence increased over time along with the global average (Figure 3b).

Prevalences in northern Africa were higher than the global average, whereas those in central, eastern and western Africa were lower. In southern Africa, compared with the global average, figures were higher in women but similar in men. Although regional time trends mirrored the global pattern in most regions, curves were steeper than the global average in recent years in northern African men and women and possibly in southern African women.

Credible intervals around the trend curves for most regions overlapped, suggesting that regional differences, while possible, were not statistically distinguishable due to limited data availability and large variability in available data (Figure 3b). Diabetes prevalence by sex was mostly similar in eastern, western and southern Africa, but might have been higher in men than in women in central Africa and higher in women than in men in northern Africa (Figure 4). Within countries, age-standardized diabetes prevalence in 2014 ranged from 4.2% (1.8–7.9) in Burundi to 16.0% (10.0–23.6) in Egypt for men, and from 4.1% (1.8–7.7) in Burundi to 19.8% (12.9–28.2) in Egypt for women (Figure 5b and Supplementary Table 2, available as Supplementary data at *IJE* online).

### Associations between diabetes and BMI

**Figure 6:**
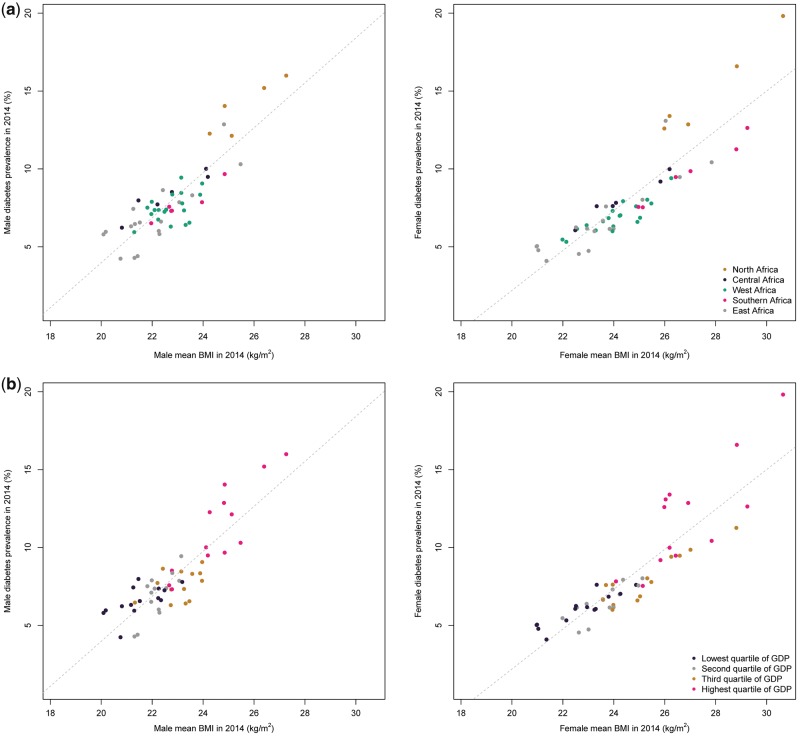
Age-standardized diabetes prevalence versus age-standardized mean body mass index in African men and women in 2014, by region and per capita gross domestic product (GDP).

Across countries, there was a strong positive association between mean BMI level and diabetes prevalence in 2014 for men and women (correlation coefficients of 0.86 and 0.88, respectively) (Figure 6). This is slightly weaker than the associations observed in 1980 (0.88 and 0.91, respectively). A moderately strong association between absolute change in BMI and relative change in diabetes was observed in women (r = 0.76), but the association was weak in men (r = 0.34).

## Discussion

Over the past three and a half decades, age-standardized mean BMI and diabetes prevalence have steadily increased across African regions, at least as steeply as the global average. There may have been an acceleration of trends for women’s BMI in all regions, and for diabetes prevalence in northern African men and women. Estimates in northern and southern Africa were mostly higher than global averages, whereas those in other regions were mostly lower. This probably reflects different stages of the epidemiological transitions and other determinants of high BMI and diabetes. Arab countries not only had the highest diabetes prevalence in Africa, but also have some of the highest worldwide prevalences,^11^ explained to some extent by differences in diet, physical (in)activity and obesity.^10^ Mean BMI was consistently higher in women than in men, whereas male-female differences in diabetes prevalence varied across regions.

The fact that average BMI increased steadily over the same period, as well as evidence of an association between BMI and diabetes in 2014, affirms the role of adiposity as a driver of diabetes across Africa. Estimates were, however, based on limited data sources, particularly in sub-Saharan Africa (SSA), with nearly 20 SSA countries having no data for diabetes.

Other investigators have estimated diabetes prevalence in Africa. Manne-Goehler and colleagues analysed individual participant data from surveys conducted in mostly 15 to 64-year-olds across 12 SSA countries between 2005 and 2015 (STEPwise approach applied in 11 surveys), and reported a median diabetes prevalence of 5% (range 2% to 14%).^12^ The International Diabetes Federation (IDF) Diabetes Atlas,^13^ using substantially fewer data sources than ours, estimated the age-standardized diabetes prevalence in 20 to 79-year-old adults in 2015 to be 3.8% (95% confidence intervals 2.1–6.7%) in SSA, and 10.7% (7.4–14.2%) in the Middle East and North Africa. In a meta-analysis of prevalence studies, published between 2000 and 2015, of diabetes among older SSA persons, the overall prevalence was 13.7% (11.3–16.3%) among ≥55 year olds.^14^ Published studies and ours have used overlapping data sources, but different time-periods, geographical coverage, diabetes definitions, population coverage, modelling/estimation strategies and the results are, therefore, not directly comparable.

Our findings of increasing diabetes prevalence across Africa between 1980 and 2014 are consistent with successive IDF estimates.^13^ However, the IDF does not estimate trends, relying instead on successive cross-sectional reports, and an earlier trend study focused on FPG.^15^ Therefore our estimates are the first consistent diabetes trend estimates for Africa, including by its regions.

Studies in populations of European origin have consistently reported higher diabetes prevalence in men than in women, with widening gaps as populations undergo the economic transition from less developed to more affluent societies.^16^^,^^17^ In the US National Health Interview Surveys, diabetes prevalence in African-Americans was higher in women than in men over the years 1963–85. However, during this same period, diabetes prevalence increased more steeply in African-American men than women.^18^ We found variable patterns of sex differences, with diabetes prevalence in 2014 being mostly similar or higher in men at a low prevalence, and higher in women at a high prevalence. Several mechanisms, reviewed elsewhere,^19^ have been suggested to explain sex differences in diabetes incidence (or the lack thereof) across populations and settings.

Five years after the adoption of the United Nations’ resolution on NCDs,^20^ and three years after the introduction of the Global NCD Action Plan,^8^ good quality nationally representative data on the prevalence of such a major NCD are still lacking in most African countries. Our findings, in consequence, were based on relatively few data points per country and on the statistical model for many countries. STEPs surveys are growing data sources for diabetes and obesity studies in Africa. Their advantages include national or regional representativeness for some, and the use of standardized questionnaires and protocols which facilitate cross-country comparisons. STEPs data, however, have limitations discussed extensively elsewhere.^12^ Furthermore, measurement of biomarkers has not been implemented across all STEPs surveys in Africa. This is unfortunate because up to two-thirds of cases remain undiagnosed and are uncovered only through biomarker measurements. Where biomarkers have been measured, the WHO recommends testing fasting venous blood glucose for diabetes.^21^ There are concerns that fasting glucose alone may not capture all cases of undiagnosed diabetes in Africans, whose early stages of the disease are probably expressed through elevated 2-h post-OGTT glucose. A study in ‘Coloured’ (i.e. mixed race) South Africans indicated that nearly 50% with undiagnosed diabetes would be missed in the absence of OGTT.^22^

Notwithstanding the challenges of implementing optimal diabetes biomarker measurements in population-based surveys, the many uncertainties around the true burden of diabetes invites cross-country efforts to generate reliable data. This, together with pleas for survey data from Africa to be more publicly available, to maximize their use for research and policy formulation,^12^^,^^23^ might serve as baseline support for the introduction of successful diabetes prevention and control efforts.

Other limitations in the interpretation of these study findings warrant consideration. Regions, as defined in this study, are general geographical entities comprising countries with substantial heterogeneities which can affect diabetes and BMI estimates. However, each region reflects existing common entities recognized by the African Union, and includes some form of concerted action on health.

The current study, however, has major strengths. This is the first time estimates have been made, from a broad African perspective, of trends in diabetes and BMI. This provides an opportunity for detailed cross-regional comparisons. Despite the limited number of data sources, estimates in this study are based on a larger amount of population-based data than previous initiatives.^12^

A high-level response to the growing diabetes threat includes the 2007 Strategy for Diabetes Prevention and Control in Africa,^5^ the 2009 Mauritius Call for Action,^6^ and the 2016 Dar es Salaam Call to Action on Diabetes and other NCDs.^7^ More generally, all countries adopted the WHO Global Action Plan for the Prevention and Control of NCDs 2013–2020.^8^ Despite the increasing awareness of the growing diabetes burden, ensuing investment and actions are not yet commensurate with the magnitude of the challenge.^24^ Going forward, the Institute of Medicine has recognized the importance of locally relevant evidence to successfully contextualize and implement prevention and control lessons from other parts of the world.^25^

Africa’s continuing urbanization offers an opportunity to develop urban environments conducive to healthy choices which could reduce obesity and diabetes risk. Tailoring the MNCH platforms which fight undernutrition to address healthy eating in general could prevent and control both under- and overnutrition. Locally relevant solutions are yet to be devised to promote effective diabetes risk reversal^26^ in Africa, where weight loss-related HIV/AIDS stigma is common. However, the platforms created to address common infectious diseases, such as HIV/AIDS and tuberculosis, offer excellent opportunities for implementing diabetes risk screening and reduction strategies.^27^ Integrating prevention and care services for common chronic infectious and non-infectious diseases in Africa^28^ could facilitate, for NCD prevention and control, the transfer of skills, and leverage some of the abundant infectious disease resources.

The current study has refined, for Africa, the previous global efforts of the NCD Risk Factor Collaboration.^10^^,^^11^ The prevalence of diabetes is substantial and possibly growing faster than elsewhere, probably triggered by increasing obesity. This study highlights the urgent need for locally relevant knowledge, supportive policies, adequately equipped health services and optimal social and physical environments to address the dual burden of rising obesity and diabetes.

## Supplementary Data

Supplementary data are available at *IJE* online.

## Funding

This study was supported by the Wellcome Trust and the South African Medical Research Council.

## Supplementary Material

Supplementary Data
