# Supplementary material for: Trends in obesity and diabetes across Africa from 1980 to 2014: an analysis of pooled population-based studies
Source: Int J Epidemiol. 2017 Jun 4;46(5):1421–32. doi: 10.1093/ije/dyx078 (PMC5837192; doi:10.1093/ije/dyx078)
Supplement: Supplementary Data [file dyx078_ije-2017-03-0321-file013.docx]

**Trends in obesity and diabetes across regions in Africa from 1980 to 2014: a pooled analysis of population-based studies**

Supplementary Data

**Supplementary Textbox 1**

**Detailed Methods**

*Overview*

We estimated trends in mean body mass index (BMI) and diabetes prevalence from 1980 to 2014, across 53 African countries which were organised geographically into five regions (Table S1). Diabetes was defined as fasting plasma glucose (FPG) ≥7.0 mmol/L or history of diagnosed diabetes or current use of insulin or oral glucose control agents. A similar definition for diabetes is used in the Global Monitoring Framework for Non-communicable Diseases (NCDs) [1]. Data from population-based surveys are more likely to measure FPG than use the 2h oral glucose tolerance test (OGTT) which is difficult even in clinical settings. Consistent with the Global Monitoring Framework for NCDs [1], we included men and women aged 18 years and older.

The study followed a number of steps described in detail elsewhere [2, 3] and summarised below.

*Data sources*

We included data sources that were representative of a national, subnational, or community population and had measured weight and height for BMI, or at least one of the following biomarkers for diabetes: FPG, OGTT or haemoglobin A1c (HbA1c). We did not use data sources that relied entirely on self-reported height and weight and/or a self-reported history of diagnosed diabetes, in order to limit the risk of bias and misdiagnosis. In particular, self-reported diabetes without a biomarker would miss undiagnosed diabetes, which forms a substantial share of all people with diabetes, especially in communities with little access to health care.

*Data re-analysis to achieve consistent mean BMI and definition of diabetes*

Anonymised individual record data were also re-analysed by the consortium or by data holders according to a common protocol. All analyses incorporated appropriate sample weights and complex survey designs when applicable.

For BMI, before calculating mean BMI, we dropped participants with implausible BMI levels (defined as BMI <10 kg/m^2^ or BMI >80 kg/m^2^; <1% of all subjects). For diabetes, 10% of the data were from sources that had reported the prevalence of diabetes based on OGTT or HbA1c but not FPG. Another 23% of data, from a previous global pooling or extracted from published reports, had used FPG but reported only mean FPG or an FPG-based diabetes prevalence using a cut-off other than 7.0 mmol/L, e.g. diabetes defined as FPG ≥7.8 mmol/L. Using these data as reported could bias the estimates because prevalence depends on how diabetes is defined [4]. To overcome the issue of incomparability, we used regressions that converted prevalence from these sources to our primary outcome, as described in detail elsewhere [3, 4]. Before deriving diabetes prevalence estimates, we dropped implausible biomarker data (FPG <2.5 mmol/L or FPG >30 mmol/L; OGTT <2 mmol/L or OGTT >30 mmol/L; HbA1c <3% or HbA1c >18%).

*Statistical methods*

Analyses were done separately for men and women to account for likely sex differences, using statistical model described in detail elsewhere [2, 3]. In summary, the model had a hierarchical structure in which estimates for each country and year were informed by its own data, data from other years in the same country and other countries, especially those in the same region. The model incorporated non-linear time trends and non-linear age patterns [5], and accounted for rural vs. urban differences, and whether data were representative at the national level or at the subnational and community vs. nationally level. The statistical models included the following covariates: national income [natural logarithm of per-capita gross domestic product adjusted for inflation and purchasing power], proportion of population living in urban areas, and a summary measure of availability of different food types for human consumption [6, 7] for mean BMI, and number of years of education, proportion of population living in urban areas, a summary measure of availability of different food types for human consumption [7], and age-standardised mean BMI for diabetes prevalence . We fitted the Bayesian model with the Markov chain Monte Carlo (MCMC) algorithm. Convergence was monitored and 5,000 post-burn-in samples were obtained from the posterior distribution of model parameters, which were in turn used to obtain the posterior distributions of mean BMI and diabetes prevalence. The reported credible intervals represent the 2.5^th^-97.5^th^ percentiles of the posterior distributions. The approach used for data analysis has been previously validated [2, 3, 8, 9].

**Supplementary Table 1: Regions in Africa**

| **Regions** | **Countries** |
| --- | --- |
| Central Africa (6) | Angola, Central African Republic, Congo, DR Congo, Equatorial Guinea, Gabon |
| Eastern Africa (17) | Burundi, Comoros, Djibouti, Eritrea, Ethiopia, Kenya, Madagascar, Malawi, Mauritius, Mozambique, Rwanda, Seychelles, Somalia, Sudan, Tanzania, Uganda, Zambia |
| North Africa (5) | Algeria, Egypt, Libya, Morocco, Tunisia |
| Southern Africa (6) | Botswana, Lesotho, Namibia, South Africa, Swaziland, Zimbabwe |
| Western Africa (19) | Benin, Burkina Faso, Cabo Verde, Cameroon, Chad, Cote d'Ivoire, Gambia, Ghana, Guinea, Guinea Bissau, Liberia, Mali, Mauritania, Niger, Nigeria, Sao Tome and Principe, Senegal, Sierra Leone, Togo |

**Supplementary Table 2: Age-standardised diabetes prevalence and mean body mass index (BMI) in men and women in 1980 and 2014 per country**

| Country | Diabetes [prevalence % (95% credibility interval)] | | | | |  | BMI [kg/m^2^ (95% credibility interval)] | | | | |
| --- | --- | --- | --- | --- | --- | --- | --- | --- | --- | --- | --- |
|  | Men | |  | Women | |  | Men | |  | Women | |
|  | 1980 | 2014 |  | 1980 | 2014 |  | 1980 | 2014 |  | 1980 | 2014 |
| Algeria | 4.7 (1.7-10.0) | 12.3 (7.4-18.8) |  | 5.6 (2.1-11.5) | 12.6 (7.9-18.1) |  | 21.9 (20.3-23.5) | 24.3 (23.0-25.4) |  | 23.7 (21.6-25.8) | 26.0 (24.7-27.2) |
| Angola | 2.6 (0.7-6.5) | 8.5 (3.8-16.2) |  | 2.9 (0.8-7.3) | 7.8 (3.6-15.1) |  | 20.3 (17.9-22.7) | 22.8 (20.4-25.1) |  | 20.1 (16.8-23.4) | 24.1 (21.0-27.2) |
| Benin | 3.1 (0.8-7.6) | 7.4 (3.7-12.4) |  | 2.7 (0.7-6.6) | 7.0 (3.6-11.6) |  | 20.5 (18.8-22.1) | 22.5 (21.5-23.6) |  | 19.5 (17.7-21.3) | 24.2 (23.4-25.0) |
| Botswana | 2.0 (0.5-5.2) | 7.6 (4.0-12.6) |  | 3.8 (1.1-9.1) | 9.5 (5.2-15.2) |  | 19.6 (17.9-21.4) | 22.7 (21.4-23.9) |  | 22.9 (20.5-25.3) | 26.4 (25.2-27.7) |
| Burkina Faso | 2.3 (0.6-6.3) | 7.4 (3.612.5) |  | 2.3 (0.6-5.9) | 5.6 (2.6-9.6) |  | 19.8 (18.0-21.6) | 22.1 (21.3-22.8) |  | 18.9 (17.4-20.4) | 22.0 (21.3-22.7) |
| Burundi | 1.0 (0.2-2.9) | 4.2 (1.8-7.9) |  | 1.5 (0.3-4.4) | 4.1 (1.8-7.7) |  | 18.9 (16.5-21.3) | 20.8 (18.5-22.9) |  | 18.2 (15.7-20.7) | 21.3 (20.1-22.6) |
| Cape Verde | 3.1 (0.8-7.6) | 9.1 (4.6-15.2) |  | 3.2 (0.9-7.8) | 8.0 (4.1-13.5) |  | 20.5 (18.8-22.2) | 24.0 (22.7-25.2) |  | 20.4 (18.2-22.7) | 25.3 (24.0-26.6) |
| Cameroon | 2.9 (0.8-7.6) | 6.5 (3.2-11.2) |  | 3.1 (0.9-7.6) | 6.9 (3.5-11.8) |  | 21.1 (19.5-22.7) | 23.5 (22.1-24.8) |  | 21.0 (19.3-22.8) | 25.0 (24.2-25.8) |
| Central African Republic | 2.9 (0.8-7.1) | 8.0 (3.5-15.4) |  | 2.9 (0.8-7.4) | 7.6 (3.5-14.8) |  | 20.0 (18.0-22.1) | 21.5 (19.9-23.0) |  | 19.7 (17.9-21.6) | 23.2 (21.7-24.9) |
| Chad | 2.8 (0.7-7.0) | 7.9 (3.9-13.8) |  | 2.5 (0.6-6.3) | 6.1 (2.9-11.0) |  | 19.8 (17.9-21.6) | 22.0 (20.4-23.5) |  | 18.7 (16.9-20.5) | 22.5 (21.2-23.9) |
| Comoros | 3.2 (0.9-7.6) | 7.9 (4.1-12.9) |  | 3.2 (0.9-7.8) | 8.0 (4.3-13.0) |  | 21.1 (19.2-22.8) | 23.1 (22.2-24.0) |  | 20.7 (18.7-22.6) | 25.1 (24.2-26.0) |
| Congo | 3.3 (0.9-7.9) | 7.7 (3.4-15.4) |  | 3.4 (1.0-8.5) | 7.6 (3.4-15.0) |  | 20.1 18.6-21.7) | 22.2 (20.5-24.0) |  | 20.3 (19.3-21.4) | 24.0 (23.0-24.9) |
| Cote d’Ivoire | 3.3 (0.9-8.4) | 7.3 (3.6-12.6) |  | 3.3 (1.0-7.9) | 6.3 (3.1-10.9) |  | 21.3 (19.6-22.9) | 23.2 (22.0-24.5) |  | 21.1 (19.4-22.7) | 24.0 (23.0-24.9) |
| Djibouti | 5.7 (2.0-11.9) | 8.6 (4.0-15.2) |  | 5.7 (2.1-11.8) | 7.6 (3.6-13.5) |  | 21.3 (19.1-23.6) | 22.4 (20.2-24.6) |  | 22.6 (19.4-25.6) | 23.7 (20.7-26.8) |
| Democratic Republic, Congo | 2.4 (0.7-5.6) | 6.2 (2.6-12.6) |  | 2.6 (0.7-6.6) | 6.1 (2.7-12.1) |  | 19.6 (17.6-21.5) | 20.8 (19.1-22.6) |  | 19.4 (17.4-21.5) | 22.5 (21.7-23.3) |
| Egypt | 6.5 (2.4-13.6) | 16.0 (10.0-23.6) |  | 8.0 (3.1-15.6) | 19.8 (12.9-28.2) |  | 24.7 (23.4-26.1) | 27.3 (26.5-28.1) |  | 26.7 (25.3-28.0) | 30.6 (30.0-31.2) |
| Equatorial Guinea | 2.5 (0.7-6.1) | 9.5 (4.3-17.7) |  | 2.9 (0.8-7.2) | 9.2 (4.3-17.3) |  | 20.1 (17.7-22.5) | 24.2 (21.7-26.6) |  | 20.3 (17.7-22.9) | 25.8 (24.5-27.1) |
| Eritrea | 2.1 (0.6-5.3) | 6.0 (2.9-10.5) |  | 2.4 (0.6-6.0) | 5.0 (2.4-9.0) |  | 18.8 (16.8-20.8) | 20.2 (19.2-21.1) |  | 18.7 (17.1-20.3) | 21.0 (20.0-21.9) |
| Ethiopia | 2.2 (0.5-5.7) | 5.8 (2.7-10.4) |  | 2.5 (0.7-5.9) | 5.0 (2.4-8.9) |  | 18.9 (17.2-20.6) | 20.1 (19.2-21.0) |  | 18.8 (16.9-20.9) | 21.0 (20.0-21.9) |
| Gabon | 3.6 (1.0-8.5) | 10.0 (4.4-19.2) |  | 3.9 (1.2-9.3) | 10.0 (4.5-19.2) |  | 21.6 (19.5-23.7) | 24.1 (22.5-25.7) |  | 21.9 (19.5-24.1) | 26.2 (25.0-27.2) |
| Gambia | 3.3 (0.9-8.2) | 9.4 (4.7-16.1) |  | 3.2 (0.9-7.6) | 7.9 (4.0-13.5) |  | 20.1 (18.4-21.6) | 23.1 (22.1-24.2) |  | 20.0 (18.2-21.7) | 24.4 (23.6-25.1) |
| Ghana | 3.0 (0.8-7.6) | 6.4 (3.1-11.0) |  | 3.0 (0.9-7.3) | 6.5 (3.4-11.2) |  | 20.9 (19.3-22.4) | 23.3 (22.2-24.5) |  | 20.7 (19.2-22.2) | 24.9 (24.0-25.8) |
| Guinea | 2.4 (0.6-6.2) | 6.7 (3.3-11.6) |  | 2.5 (0.7-6.4) | 6.0 (3.1-10.3) |  | 20.2 (18.4-22.1) | 22.2 (20.8-23.6) |  | 19.6 (17.7-21.6) | 23.3 (22.4-24.2) |
| Guinea Bissau | 2.6 (0.7-6.6) | 7.4 (3.7-12.6) |  | 2.8 (0.8-6.9) | 6.8 (3.4-11.8) |  | 20.3 (18.1-22.5) | 22.3 (20.1-24.4) |  | 20.1 (17.7-22.4) | 23.8 (22.5-25.1) |
| Kenya | 2.1 (0.6-5.2) | 5.8 (2.9-10.0) |  | 2.7 (0.8-6.7) | 6.2 (3.1-10.4) |  | 20.0 (18.4-21.4) | 22.3 (20.5-24.1) |  | 20.3 (18.9-21.8) | 24.0 (22.8-25.1) |
| Lesotho | 2.3 (0.5-5.7) | 7.3 (3.5-12.8) |  | 4.8 (1.4-11.6) | 9.8 (5.0-16.3) |  | 20.1 (18.4-21.9) | 22.7 (22.0-23.5) |  | 24.4 (22.6-26.1) | 27.0 (26.1-27.8) |
| Liberia | 4.0 (1.2-9.2) | 7.8 (4.1-12.9) |  | 3.5 (1.1-8.2) | 7.6 (4.1-12.7) |  | 21.9 (20.2-23.7) | 23.2 (22.5-23.9) |  | 21.0 (18.9-23.2) | 24.9 (23.9-25-7) |
| Libya | 7.5 (3.0-14.8) | 15.2 (9.5-22.5) |  | 9.1 (3.7-17.1) | 16.6 (10.7-23.8) |  | 24.3 (22.7-25.9) | 26.4 (25.3-27.4) |  | 27.1 (24.9-29.2) | 28.8 (27.7-30.0) |
| Madagascar | 2.4 (0.7-6.0) | 6.3 (3.1-10.8) |  | 2.6 (0.8-6.2) | 4.8 (2.4-8.3) |  | 19.8 (18.2-21.5) | 21.2 (19.6-22.7) |  | 19.6 (17.9-21.5) | 21.0 (20.0-22.0) |
| Malawi | 2.3 (0.6-5.8) | 6.6 (3.3-11.4) |  | 2.7 (0.8-6.8) | 6.0 (3.1-9.9) |  | 20.3 (18.6-21.9) | 22.3 (21.3-23.4) |  | 20.1 (18.6-21.6) | 23.2 (22.4-24.0) |
| Mali | 3.0 (0.8-7.5) | 8.4 (4.0-14.5) |  | 2.6 (0.7-6.4) | 6.4 (3.0-11.5) |  | 20.5 (18.9-22.1) | 22.8 (21.3-24.3) |  | 19.1 (17.4-20.8) | 22.9 (21.9-23.8) |
| Mauritania | 3.3 (0.9-8.0) | 8.5 (4.1-14.6) |  | 4.0 (1.2-9.3) | 9.4 (4.7-15.8) |  | 20.9 (19.0-22.8) | 23.1 (21.5-24.7) |  | 22.4 (20.2-24.4) | 26.2 (24.7-27.8) |
| Mauritius | 7.1 (3.0-13.1) | 12.9 (7.2-20.1) |  | 8.9 (4.0-15.9) | 13.1 (7.5-20.1) |  | 21.9 (20.9-23.0) | 24.8 (23.8-25.8) |  | 23.3 (22.2-24.5) | 26.0 (25.0-27.1) |
| Morocco | 4.9 (1.7-10.3) | 14.0 (8.4-21.5) |  | 5.4 (2.0-11.5) | 13.4 (8.1-20.5) |  | 22.2 (20.7-23.7) | 24.8 (23.3-26.4) |  | 23.5 (22.0-25.1) | 26.2 (24.9-27.5) |
| Mozambique | 2.3 (0.6-5.7) | 6.6 (3.3-11.4) |  | 2.6 (0.7-6.6) | 6.2 (3.2-10.6) |  | 19.7 (18.0-21.4) | 21.5 (20.2-22.8) |  | 19.9 (18.0-21.8) | 22.9 (22.0-23.9) |
| Namibia | 2.6 (0.7-6.1) | 7.3 (3.5-12.6) |  | 3.8 (1.2-8.9) | 7.5 (3.9-12.7) |  | 20.5 (18.8-22.2) | 22.8 (21.5-24.0) |  | 22.7 (21.1-24.3) | 25.1 (24.4-25.9) |
| Niger | 2.5 (0.6-6.4) | 5.9 (2.7-10.9) |  | 2.5 (0.7-6.4) | 5.3 (2.4-9.5) |  | 19.8 (18.1-21.5) | 21.3 (20.0-22.5) |  | 19.1 (17.5-20.7) | 22.1 (21.1-23.0) |
| Nigeria | 2.6 (0.7-6.6) | 6.3 (3.1-10.6) |  | 3.2 (0.9-7.7) | 6.0 (3.1-10.3) |  | 20.5 (19.3-21.7) | 22.7 (21.9-23.3) |  | 21.3 (20.1-22.6) | 23.9 (23.3-24.6) |
| Rwanda | 1.1 (0.2-3.1) | 4.3 (1.8-8.0) |  | 1.6 (0.3-4.7) | 4.5 (2.1-8.1) |  | 19.0 (17.2-20.9) | 21.3 (20.6-22.0) |  | 19.0 (17.0-21.0) | 22.6 (21.8-23.4) |
| Sao Tome and Principe | 4.3 (1.3-10.1) | 8.3 (4.1-14.2) |  | 4.1 (1.3-9.6) | 7.8 (3.9-13.2) |  | 21.6 (19.9-23.3) | 23.9 (23.0-24.8) |  | 21.9 (19.5-24.1) | 25.4 (24.4-26.3) |
| Senegal | 3.6 (1.0-8.4) | 7.5 (3.7-12.9) |  | 4.0 (1.2-9.2) | 7.3 (3.7-12.6) |  | 20.1 (18.6-21.5) | 21.8 (20.9-22.7) |  | 21.3 (20.3-22.3) | 23.9 (23.0-24.8) |
| Seychelles | 5.0 (1.7-10.7) | 10.3 (5.5-16.7) |  | 6.4 (2.4-12.8) | 10.4 (5.6-16.7) |  | 22.1 (20.9-23.3) | 25.5 (24.7-26.2) |  | 24.6 (23.3-25.9) | 27.8 (27.0-28.7) |
| Sierra Leone | 3.2 (0.9-7.8) | 7.1 (3.5-12.1) |  | 3.5 (1.1-8.2) | 6.6 (3.3-11.5) |  | 20.6 (18.9-22.4) | 22.0 (21.3-22.7) |  | 21.1 (18.9-23.3) | 23.6 (22.8-24.3) |
| Somalia | 3.0 (0.9-7.2) | 7.4 (3.6-12.8) |  | 3.2 (1.0-7.5) | 6.2 (3.1-11.1) |  | 20.3 (18.1-22.5) | 21.3 (19.0-23.5) |  | 20.6 (17.5-23.7) | 22.5 (19.5-25.6) |
| South Africa | 4.8 (1.6-10.2) | 9.7 (4.9-16.3) |  | 7.7 (2.9-15.5) | 12.6 (6.8-20.2) |  | 22.8 (21.5-24.1) | 24.8 (24.3-25.4) |  | 26.7 (25.1-28.4) | 29.2 (28.7-29.8) |
| Sudan | 3.1 (0.9-7.3) | 8.3 (4.3-13.7) |  | 4.3 (1.4-9.8) | 9.5 (5.0-15.6) |  | 21.1 (19.5-22.7) | 23.6 (22.2-24.9) |  | 23.0 (20.7-25.2) | 26.6 (25.1-28.0) |
| Swaziland | 2.7 (0.7-6.5) | 7.9 (4.0-13.4) |  | 5.1 (1.6-11.7) | 11.3 (6.1-18.1) |  | 21.1 (19.3-22.8) | 24.0 (22.7-25.2) |  | 24.9 (22.4-27.4) | 28.8 (27.4-30.2) |
| Tanzania | 2.3 (0.6-5.7) | 6.0 (3.1-9.9) |  | 2.6 (0.8-6.3) | 6.1 (3.3-9.9) |  | 20.5 (18.9-22.0) | 22.3 (21.5-23.0) |  | 20.4 (19.0-21.9) | 23.8 (23.2-24.5) |
| Togo | 3.1 (0.9-7.8) | 7.2 (3.8-12.3) |  | 2.8 (0.8-7.1) | 7.0 (3.6-11.6) |  | 20.5 (18.8-22.3) | 22.5 (21.5-23.5) |  | 19.8 (17.8-21.7) | 24.1 (21.8-26.4) |
| Tunisia | 4.8 (1.8-9.6) | 12.1 (7.4-18.3) |  | 6.0 (2.3-11.8) | 12.9 (7.9-19.0) |  | 22.1 (20.7-23.5) | 25.1 (24.0-26.2) |  | 24.6 (22.9-26.4) | 31.5 (28.9-34.0) |
| Uganda | 1.4 (0.3-3.9) | 4.4 (2.1-7.8) |  | 2.1 (0.5-5.5) | 4.7 (2.3-8.2) |  | 19.3 (17.6-21.0) | 21.4 (20.6-22.2) |  | 20.0 (18.3-21.7) | 23.0 (22.1-23.9) |
| Zambia | 3.2 (1.0-7.3) | 6.5 (3.3-11.0) |  | 4.0 (1.3-8.9) | 6.7 (3.5-11.1) |  | 19.8 (18.1-21.5) | 21.3 (19.9-22.7) |  | 21.4 (20.0-22.9) | 23.6 (22.2-26.1) |
| Zimbabwe | 2.9 (0.8-7.0) | 6.5 (3.1-11.6) |  | 5.1 (1.6-11.6) | 7.6 (3.9-12.7) |  | 20.9 (19.6-22.2) | 22.0 (21.1-22.9) |  | 24.3 (22.9-25.9) | 25.0 (24.1-25.8) |

**References**

1. World Health Organization. Global action plan for the prevention and control of noncommunicable diseases 2013-2020 World Health Organization, Geneva, Switzerland. 2013 [Available from: <http://apps.who.int/iris/bitstream/10665/94384/1/9789241506236_eng.pdf?ua=1>.

2. NCD Risk Factor Collaboration. Trends in adult body-mass index in 200 countries from 1975 to 2014: a pooled analysis of 1698 population-based measurement studies with 19.2 million participants. Lancet 2016; 387:1377-1396.

3. NCD Risk Factor Collaboration. Worldwide trends in diabetes since 1980: a pooled analysis of 751 population-based studies with 4.4 million participants. Lancet 2016; 387:1513-1530.

4. NCD Risk Factor Collaboration. Effects of diabetes definition on global surveillance of diabetes prevalence and diagnosis: a pooled analysis of 96 population-based studies with 331 288 participants. Lancet Diabetes Endocrinol 2015; 3:624-637.

5. Singh GM, Danaei G, Pelizzari PM, et al. The age associations of blood pressure, cholesterol, and glucose: analysis of health examination surveys from international populations. Circulation 2012; 125:2204-2211.

6. Danaei G, Singh GM, Paciorek CJ, et al. The global cardiovascular risk transition: associations of four metabolic risk factors with national income, urbanization, and Western diet in 1980 and 2008. Circulation 2013; 127:1493-1502, 1502e1491-1498.

7. Ezzati M, Riboli E. Behavioral and dietary risk factors for noncommunicable diseases. N Engl J Med 2013; 369:954-964.

8. Danaei G, Finucane MM, Lu Y, et al. National, regional, and global trends in fasting plasma glucose and diabetes prevalence since 1980: systematic analysis of health examination surveys and epidemiological studies with 370 country-years and 2.7 million participants. Lancet 2011; 378:31-40.

9. Finucane MM, Stevens GA, Cowan MJ, et al. National, regional, and global trends in body-mass index since 1980: systematic analysis of health examination surveys and epidemiological studies with 960 country-years and 9.1 million participants. Lancet 2011; 377:557-567.
